# Supplementary material for: DMRforPairs: identifying Differentially Methylated Regions between unique samples using array based methylation profiles
Source: BMC Bioinformatics. 2014 May 15;15:141. doi: 10.1186/1471-2105-15-141 (PMC4046028; doi:10.1186/1471-2105-15-141)
Supplement: Additional file 2 — DMRforPairs output for the comparison of A431-MCF7 and NA17018-NA17105. Please start from the HTML files in each folder. Available via the BMC Bioinformatics website. [file 1471-2105-15-141-S2.zip › 1394847754114233_MOESM2_ESM/A431_MCF7/figures/33228.pdf]

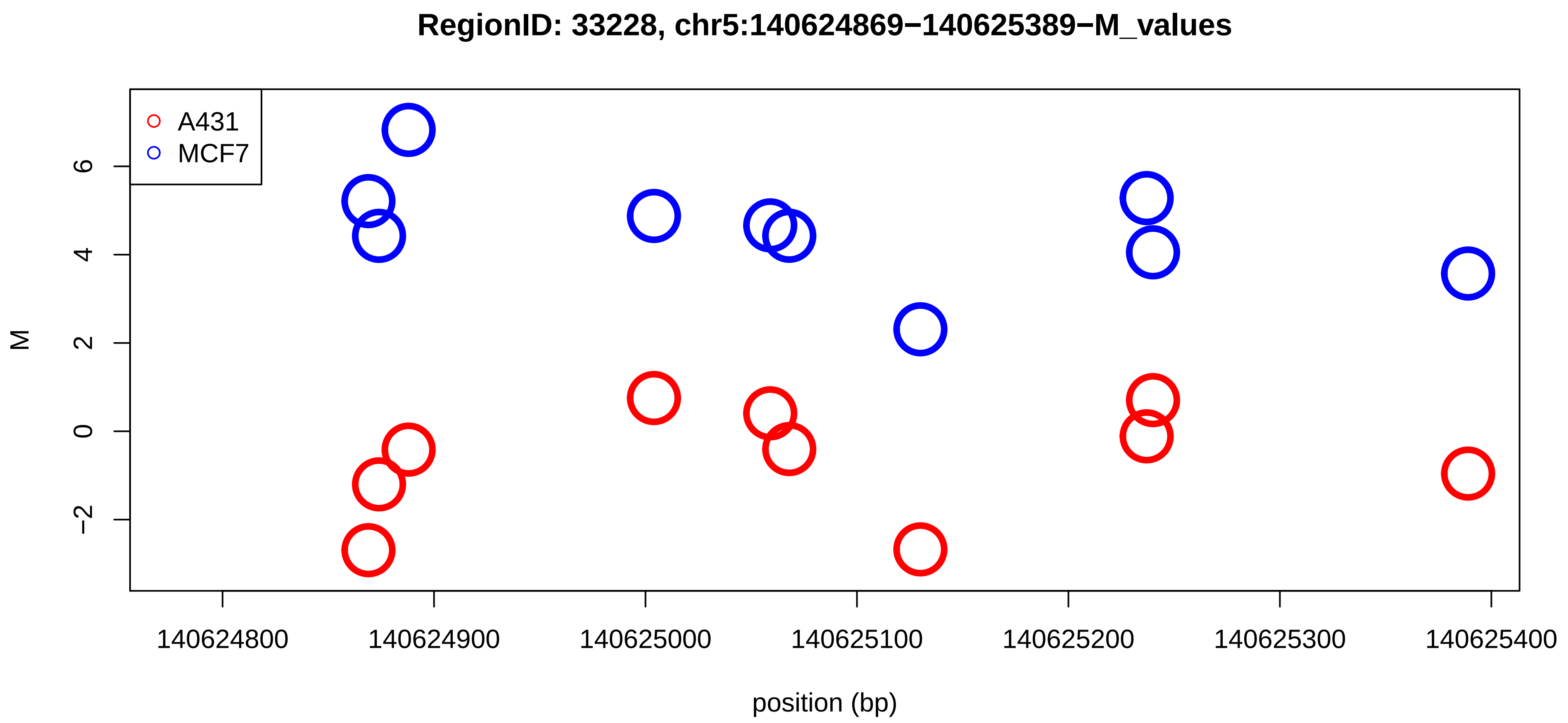
RegionID: 33228, chr5:140624869−140625389−M_values
A431
MCF7
M
−2
0
2
4
6
140624800
140624900
140625000
140625100
140625200
140625300
140625400
position (bp)

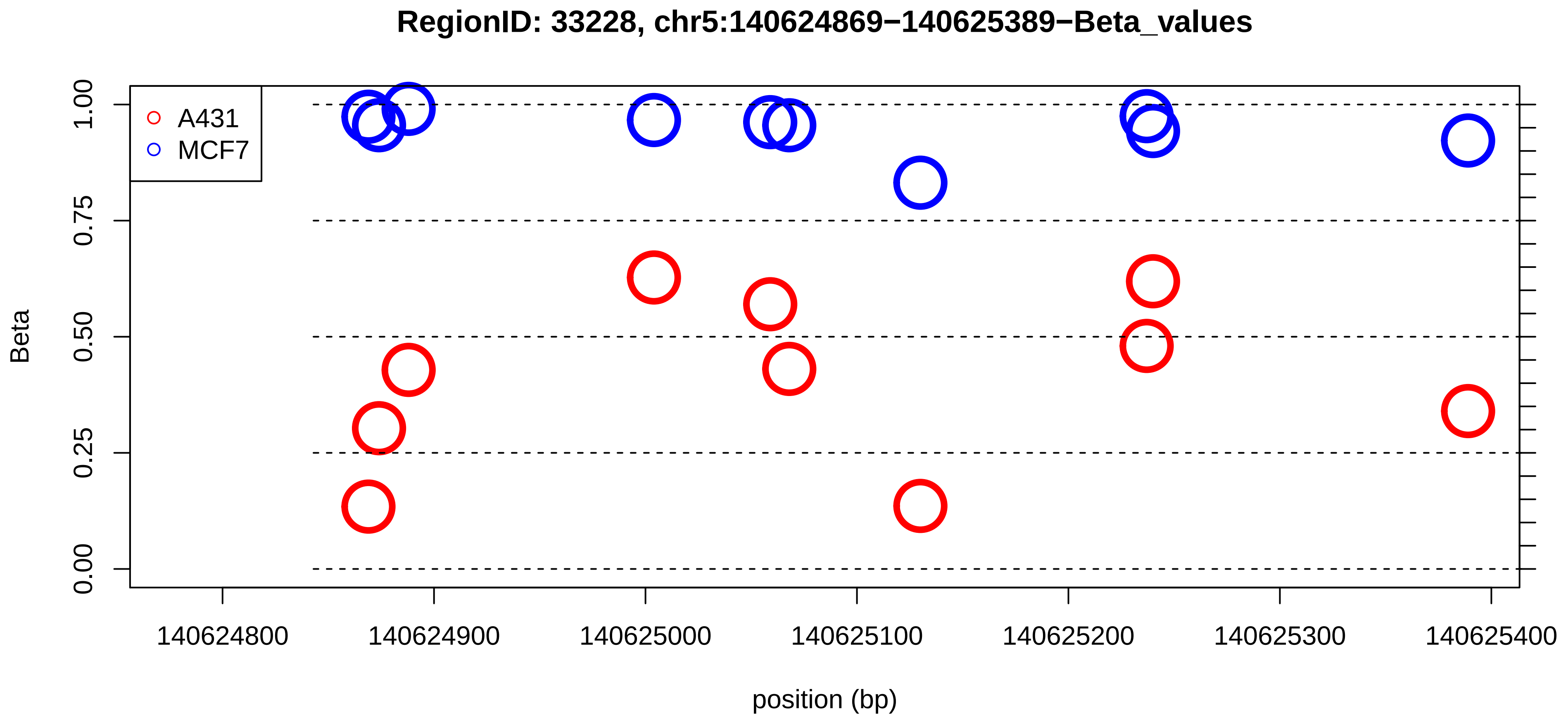
RegionID: 33228, chr5:140624869−140625389−Beta_values
A431
MCF7
Beta
0.00
0.25
0.50
0.75
1.00
140624800
140624900
140625000
140625100
140625200
140625300
140625400
position (bp)

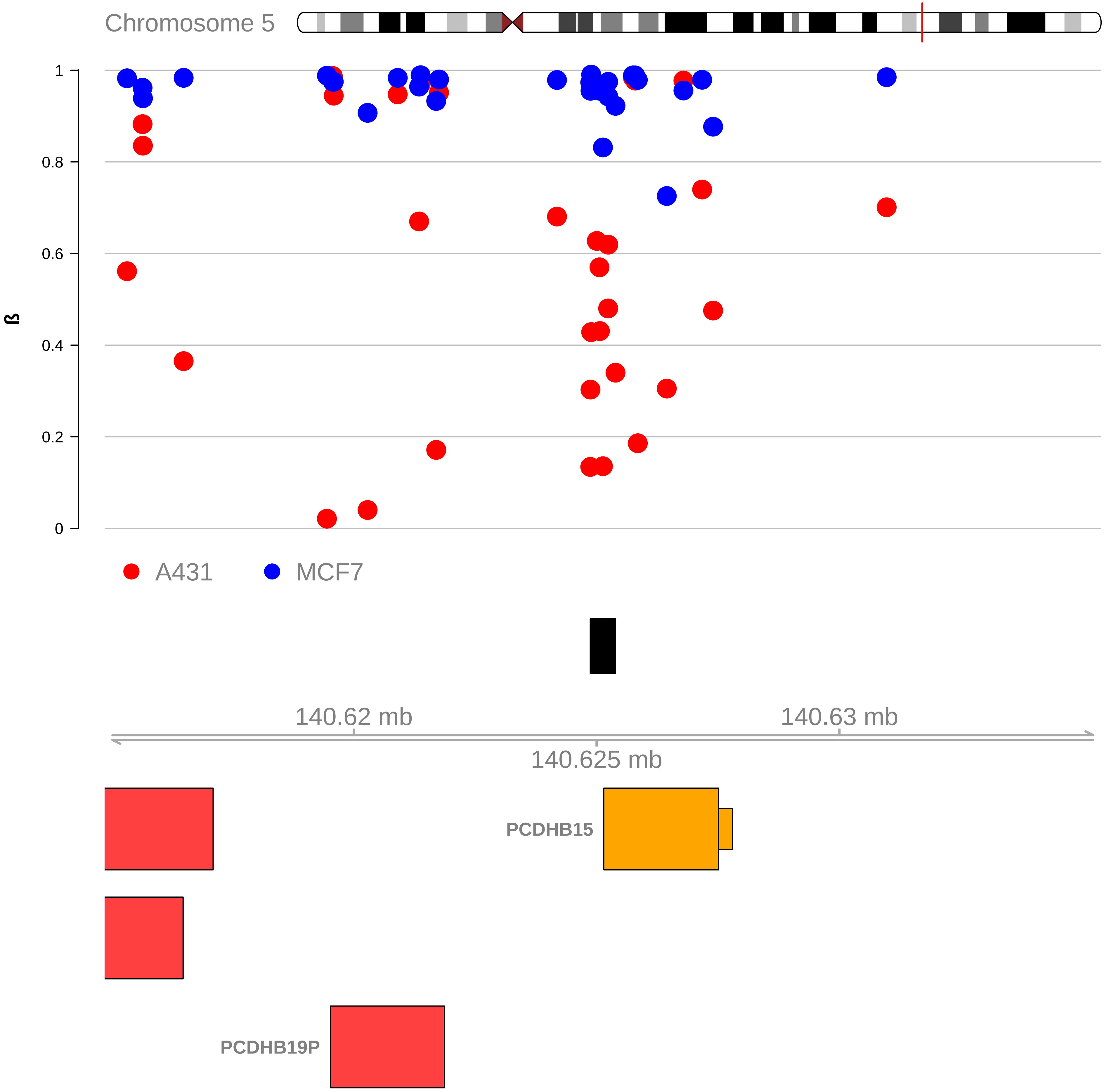

Chromosome 5
1
0.8
0.6
0.4
0.2
0
ß
A431
MCF7
140.62 mb
140.63 mb
140.625 mb
PCDHB15
PCDHB19P
